# Plausibility of super high-flux dialyzer reuse in maintenance hemodialysis

**DOI:** 10.3389/fmed.2025.1655099

**Published:** 2025-10-22

**Authors:** Piyapun Prapunwatana, Thana Thongsricome, Asada Leelahavanichkul, Patcharin Injan, Pajaree Chariyavilaskul, Paweena Susantitaphong, Yingyos Avihingsanon, Somchai Eiam-Ong, Khajohn Tiranathanagul

**Affiliations:** ^1^Division of Nephrology, Department of Medicine, Faculty of Medicine, Chulalongkorn University, Bangkok, Thailand; ^2^Department of Medicine, Chulabhorn Hospital, Chulabhorn Royal Academy, Bangkok, Thailand; ^3^Department of Physiology, Faculty of Medicine, Chulalongkorn University, Bangkok, Thailand; ^4^Center of Excellence in Translational Research in Inflammation and Immunology, Faculty of Medicine, Chulalongkorn University, Bangkok, Thailand; ^5^Center of Excellence in Clinical Pharmacokinetics and Pharmacogenomics, Department of Pharmacology, Faculty of Medicine, Chulalongkorn University, Bangkok, Thailand

**Keywords:** super high-flux dialyzer, medium cut-off dialyzer, dialyzer reuse, hemodialysis, green nephrology

## Abstract

**Background:**

Dialyzer reuse in traditional hemodialysis (HD) has been demonstrated to reduce medical waste and manufacturing costs compared to a single-use strategy. HD techniques nowadays have increasingly shifted to convective therapies, such as HD with super high-flux dialyzers (SHF), to remove larger uremic toxins and improve outcomes. However, studies on the reuse of SHF are lacking. Successful reuse of SHF may lower the economic and environmental burdens while maintaining superior clinical outcomes.

**Objective:**

To compare the removal of uremic toxins and safety after reuse of SHF.

**Methods:**

ELISIO-21 HX SHF were reprocessed with peracetic acid for up to 15 reuse times in stable thrice-a-week HD patients in King Chulalongkorn Memorial Hospital. The reduction ratio (RR) or clearance of β2-microglobulin (β2M, 11.8 kDa), α1-microglobulin (α1M, 31 kDa), λ-free light chain (λFLC, 45 kDa), and protein-bound indoxyl sulfate were compared between the 1st, 2nd, 5th, 10th, and 15th reuse times. Dialysate albumin loss and the change in serum albumin were assessed.

**Results:**

A total of 15 dialyzers were investigated from 5 patients. The β2M clearance and RR were comparable between the 1st and 15th use (127.2 ± 18.3 mL/min vs. 114.4 ± 17.2 mL/min, *p*-value 0.93 and 85.5% ± 5.9% vs. 82.5% ± 3.5%, *p*-value 1.00, respectively). The λ-FLC and indoxyl sulfate RR significantly reduced while α1M RR remained unchanged across the study period. Dialysate albumin loss decreased significantly from 1.01 g during the 1st use to 0.19 g during the 2nd and 0.06 g during the 5th use (*p*-value < 0.001). However, there was no statistically significant change in serum albumin. No adverse effect was observed throughout the study.

**Conclusion:**

Super high-flux dialyzers reuse is a safe and promising method to reduce medical waste and manufacturing costs while maintaining the benefits of this novel HD technique. We also suggest appropriate cut-off points for the change in RR of the middle-molecule uremic toxin after reuse to prevent significant impairment in solute clearances.

## Introduction

End-stage kidney disease requiring dialysis has increased in prevalence by up to 2.5 times in the past 5 years (1). Currently, the life expectancy of dialysis patients is longer than in the past, but it is not comparable to that of the general population. This might, at least in part, be explained by the accumulation of middle-molecule uremic toxins that can result in uremic complications and lead to higher morbidity and mortality (2). Current advanced hemodialysis (HD) techniques that can effectively eliminate middle-molecule uremic toxins include high-volume online hemodiafiltration (HDF) and HD using super high-flux dialyzers (SHF), so-called expanded hemodialysis (HDx). Both modalities have been demonstrated to pose a comparable removal of middle-molecule uremic toxins such as β2-microglobulin (β2M, 11.8 kDa), α1-microglobulin (α1M, 31 kDa), and λ-free light chain (λ-FLC, 45 kDa). These removals are higher than those of conventional high-flux HD (3–7). Although online HD, which has been widely used for more than two decades, has numerous mechanistic studies and clinical trials demonstrating superior removal of middle uremic toxins and decreased mortality compared to conventional high-flux HD, it requires a specialized dialysis machine with additional programming and staff training, which are not available in many hemodialysis centers (8–12). HDx exploits novel SHF or medium cut-off (MCO) dialyzers, which have larger pore sizes, to effectively remove larger middle-molecule compounds with conventional and simpler HD machines (8). Some studies even demonstrated that HDx tends to remove middle-molecule uremic toxins more effectively than online HDF (6, 7, 13). This makes HDx more practical than online HDF for large-scale applications with comparable outcomes. However, the cost of SHF is still high and there is no manufacturer approval for dialyzer reuse, which are major obstacles to the widespread application of HDx, especially in countries with limited resources.

Dialyzer reuse is a protocol developed to permit repeated HD with the same dialyzer for up to 12–20 times with careful monitoring to prevent adverse effects in the patients. The purposes of dialyzer reuse include reducing the cost of new dialyzers for every HD session, preventing the shortage of new dialyzer supplies, and possibly reducing medical waste associated with the dialyzer discard process (14). These purposes are more important for the dialyzer with higher production costs and a more limited supply, such as the SHF. However, studies on dialyzer reuse to date have been restricted to conventional low- and high-flux dialyzers. Indeed, the reuse or reprocessing of these conventional dialyzers is widely adopted in Thailand because of economic benefit. Theoretically, the reprocessing of SHF with adequate surveillance of efficacy change is feasible and may endorse widespread implementation of HDx to improve patient outcomes while compromising an environmental burden, complying with the green nephrology policy (15). The safety issue is another concern for dialyzer reprocessing, both from inadequate dialysis if the solute clearance is significantly impaired and from the residual chemical used in the sterilizing process that could enter the patient’s circulation in subsequent dialysis. Therefore, we conducted this study to compare the efficacy and safety of hemodialysis with reused SHF.

## Methodology

### Study design

A single-center prospective cohort study was conducted in prevalent thrice-a-week HD patients at the Division of Nephrology, King Chulalongkorn Memorial Hospital, Bangkok, Thailand. The inclusion criteria were aged 18–90 years, achieving a single pool Kt/V of at least 1.2, having residual renal function less than 100 mL/day, using arteriovenous fistula or graft as vascular access, having dialysis blood flow rate (BFR) of at least 400 mL/min, and being hemodynamically stable for at least 2 weeks. Exclusion criteria were active cardiovascular disease, active cancer, decompensated liver cirrhosis, pregnancy, baseline serum albumin less than 3.5 g/dL, and having a contraindication to heparin. The eligible patients would then switch to HDx with Fresenius 5008H (Fresenius Medical Care, Bad Homburg, Germany) HD machines and SHF ELISIO-21HX (Nipro Corporation, Osaka, Japan), a polyethersulfone membrane with an ultrafiltration coefficient of 82 mL/h/mmHg, sterilized with gamma irradiation, and a surface area of 2.1 m^2^. The dialysis prescriptions were 4 h per session, a BFR of 400 mL/min, and a dialysate flow rate of 800 mL/min. The dialysate water was ultrapure according to the Association for the Advancement of Medical Instrumentation (AAMI) standard (16). Unfractionated heparin was used as an anticoagulant during HDx. Net fluid removal depended on the patient’s dry weight, as judged by physicians. The characteristics of the dialyzer are summarized in Table 1.

**TABLE 1:** Characteristics of the super high-flux dialyzer used in the study.

Dialyzer characteristics	Detail
Dialyzer model	ELISIO-21HX
Membrane	Polyether sulfone
Effective surface area (m^2^)	2.1
Ultrafiltration coefficient (ml/h/mmHg)	82
**Clearance (mL/min)**	
Urea	358
Creatinine	334
Phosphate	314
Vitamin B12	240
Myoglobin	148

### Dialyzer reprocessing

According to the AAMI standard, all dialyzers in our center were manually rinsed and cleaned with reverse osmosis water until no visible blood remained. The rinsing process is different from that for conventional high-flux dialyzers, in that the header of SHF is not designed to be able to dissociate from the dialyzer body for the rinsing of hollow fibers. Therefore, specialized devices from the manufacturer were used to rinse the water into the blood inlet to clean the blood clot in the hollow fibers (Supplementary Material 1). The SHF were then washed and sterilized with 5% peracetic acid using the KIDNY-KLEEN Dialyzer Reprocessor, Model Compact II (Meditop Co., Ltd., Thailand) in a similar manner to the reprocessing of conventional dialyzers. The total cell volume (TCV) of each dialyzer was measured by the machine during every reprocessing cycle. Dialyzers were discarded if the TCV declined to less than 80% of the baseline value, if residual clots were observed, or after a maximum of 15 reuses (16). The total time for reprocessing is 10–15 min for each dialyzer. Apart from the costs of dialyzers and the automated reprocessing machine, there was no additional processing cost or specialized personnel since the reprocessing process is similar to the routine dialyzer reuse in our center.

### Measurement of uremic toxin removal

The performance of dialyzers was evaluated during the 1st (baseline), 2nd, 5th, 10th, and 15th use. During dialysis, four blood samples were collected to measure for selected uremic toxins: (1) pre-dialysis blood sample from the arterial needle; (2) blood from the arterial port of the dialysis circuit at 1st hour of HD; (3) blood from the venous port of the dialysis circuit at 1st hour of HD; and (4) post-dialysis blood sample from the arterial port of the dialysis circuit. The clearance of β2M and the RR of β2M, α1M, λ-FLC, and indoxyl sulfate were calculated.

The clearance (K) of β2M was calculated using the following equations (17):


$$\begin{array}{c} K=\frac{Q_{b\,p}\times C_{i\,n}-\left(Q_{b\,p}-Q_{u\,f}\right)\;C_{o\,u\,t}}{C_{i\,n}}\\ Q_{b\,p}=Q_{b}\times \left(1-H\,c\,t\right)\times \left(1-0.0107\times P\,t\right) \end{array}$$

C_*in*_ and C_*out*_ refer to the solute concentrations at the dialyzer inlet and the dialyzer outlet, respectively. Q_*uf*_ refers to the ultrafiltration rate in mL/min and Q_*bp*_ refers to plasma water flow in mL/min, which is calculated from BFR (Q_*b*_), hematocrit (Hct), and total protein (Pt) in g/L.


$$\begin{array}{c} RR\left(\%\right)=\left(1-\frac{C_{p\,o\,s\,t-c\,r\,r}}{C_{p\,r\,e}}\right)\times 100\\ C_{p\,o\,s\,t-c\,r\,r}=\frac{C_{p\,o\,s\,t}}{1+\left[B\,W_{p\,r\,e}+B\,W_{p\,o\,s\,t}/0.2\,\left(B\,W_{p\,o\,s\,t}\right)\right]} \end{array}$$

The reduction ratio (RR) of uremic toxins was calculated using the following equations (13):

C_*pre*_ and C_*post*_ refer to solute concentrations at pre- and post-dialysis, respectively. C_*post–crr*_ is the corrected C_*post*_ with pre- and post-dialysis body weight (BW_*pre*_ and BW_*post*,_ respectively).

### Measurement of albumin loss in the dialysate

Dialysate was collected using an infusion pump connected to the outlet line when the dialysate removal rate was set to 400 ml/h. The albumin loss into the dialysate was evaluated at the 1st, 2nd, 5th, 10th, and 15th uses. Kt/V, serum albumin, and other routine laboratory tests were also measured monthly.

### Statistical analysis

Categorical data and continuous data were presented in frequency (percentage) and mean ± standard deviation, respectively. A pairwise comparison of a mixed model for repeated measures was used to compare the difference in quantitative data at each time point and the baseline level. A *p*-value of less than 0.05 was considered statistically significant. Statistical analysis was performed using IBM SPSS Statistics version 28 for Windows.

## Results

Five patients with end-stage renal disease were enrolled. The mean age was 58.6 ± 7.1 years; three were male and two were female. The mean duration of hemodialysis was 4.0 ± 7.9 years and the mean Kt/V was 2.83 ± 0.8. Vascular access was arteriovenous fistulas in four patients and an arteriovenous graft in one patient. The underlying causes of end-stage renal disease were diabetes mellitus (*n* = 2), hypertension (*n* = 1), and unknown etiology (*n* = 2). Two patients had a history of previous kidney transplantation. The baseline clinical characteristics are presented in Table 2.

**TABLE 2:** Baseline characteristics of the patients included in the study.

Patient characteristics		*N* = 5
Age, mean ± SD (years)		58.6 ± 7.1
Sex	*n* (%) – male	3 (60)
	*n* (%) - female	2 (40)
Duration of hemodialysis, mean ± SD (years)		4.0 ± 7.9
Dry weight, mean ± SD (kg)		52.7 ± 12.9
Dialysis adequacy (Kt/V), mean ± SD		2.83 ± 0.8
**Previous dialysis modality**		
Hemodiafiltration, *n* (%)		5 (100)
**Vascular access**		
AVF, *n* (%)		4 (80)
AVG, *n* (%)		1 (20)
**Ultrafiltration rate (mL/min), mean (range)**		1027 (760–1255)
**Etiology of ESRD**		
Diabetic nephropathy, *n* (%)		2 (40)
Hypertensive nephrosclerosis, *n* (%)		1 (20)
Unknown, *n* (%)		2 (40)
**Laboratory parameters, mean ± SD**		
Hemoglobin (g/dL)		11.7 ± 0.7
Platelet count (×10^3^/μL)		199 ± 63
White blood cell count (cells/μL)		7016 ± 2179
Urea (mg/dL)		49.6 ± 13.4
Creatinine (mg/dL)		9.8 ± 1.4
Calcium (mg/dL)		9.2 ± 0.8
Phosphate (mg/dL)		3.4 ± 1.2
Serum albumin (g/L)		3.9 ± 0.2
Erythrocyte sedimentation rate (mm/h)		17.2 ± 13.0
β2-microglobulin (mg/L)		30.3 ± 5.7
α1-microglobulin (mg/L)		56.7 ± 11.4171.6±
λ-free light chain (mg/L)		37.2
Free indoxyl sulfate (mg/dL)		41.7 ± 13.5
Total indoxyl sulfate (mg/dL)		2.6 ± 1.5

Categorical data and continuous data are presented in frequency (percentage), mean ± standard deviation, respectively.

**FIGURE 1:**
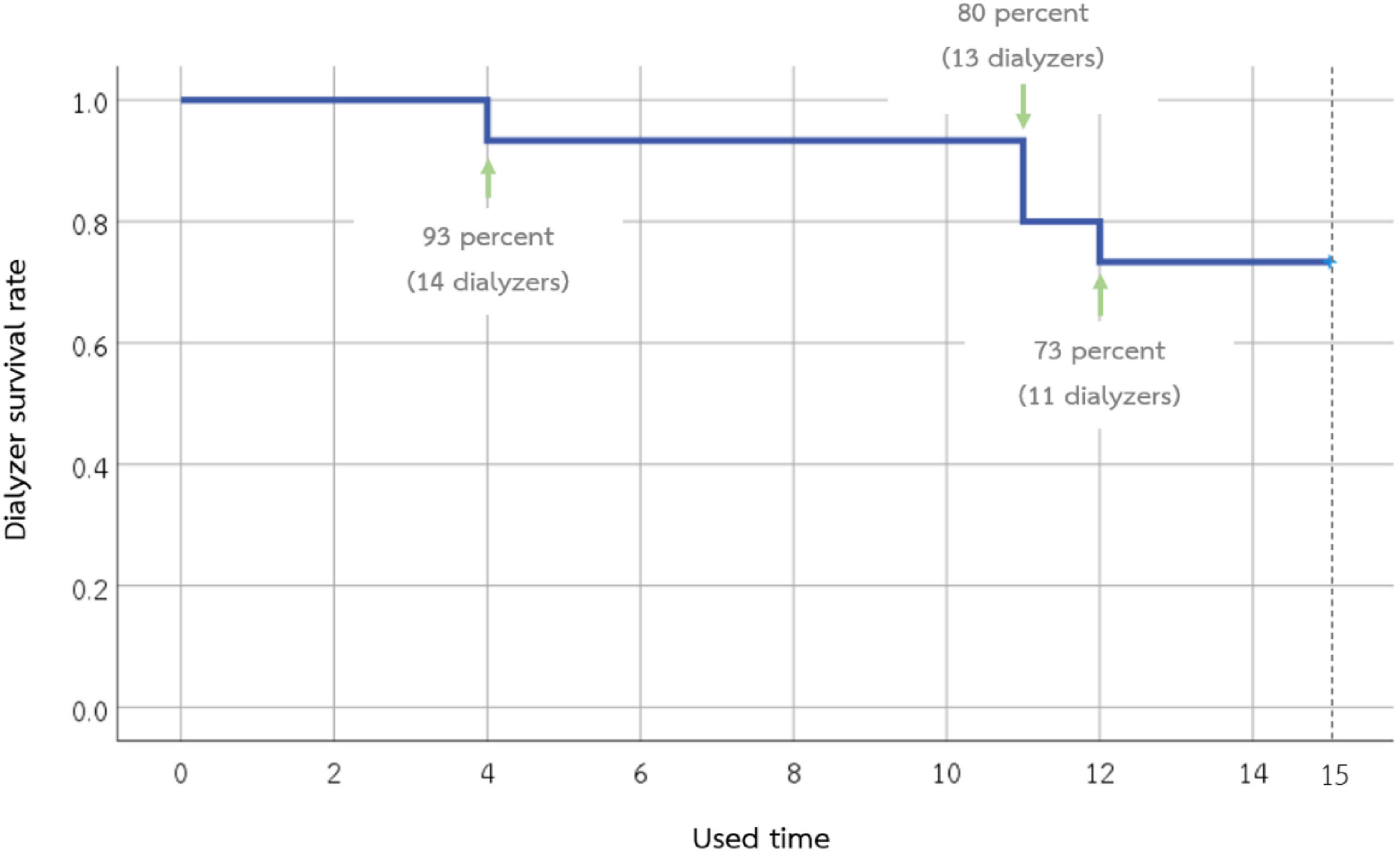
Survival of the 15 super high-flux dialyzers included in the study. Eleven dialyzers can be used until the 15th time. Dialyzers that were discarded early had residual blood clots observed during the reprocessing.

In this study, a total of 15 dialyzers were investigated. The number of dialyzers completed on the 1st, 2nd, 5th, 10th, and 15th used was 15, 15, 14, 14, and 11, respectively (Figure 1). Dialyzers that were discarded early had residual blood clots observed during the reprocessing. One dialyzer had a TCV of less than 80% after the 15th reuse, while the remaining TCV measurements in this study were more than 80% of the baseline.

### β2M removal

**FIGURE 2:**
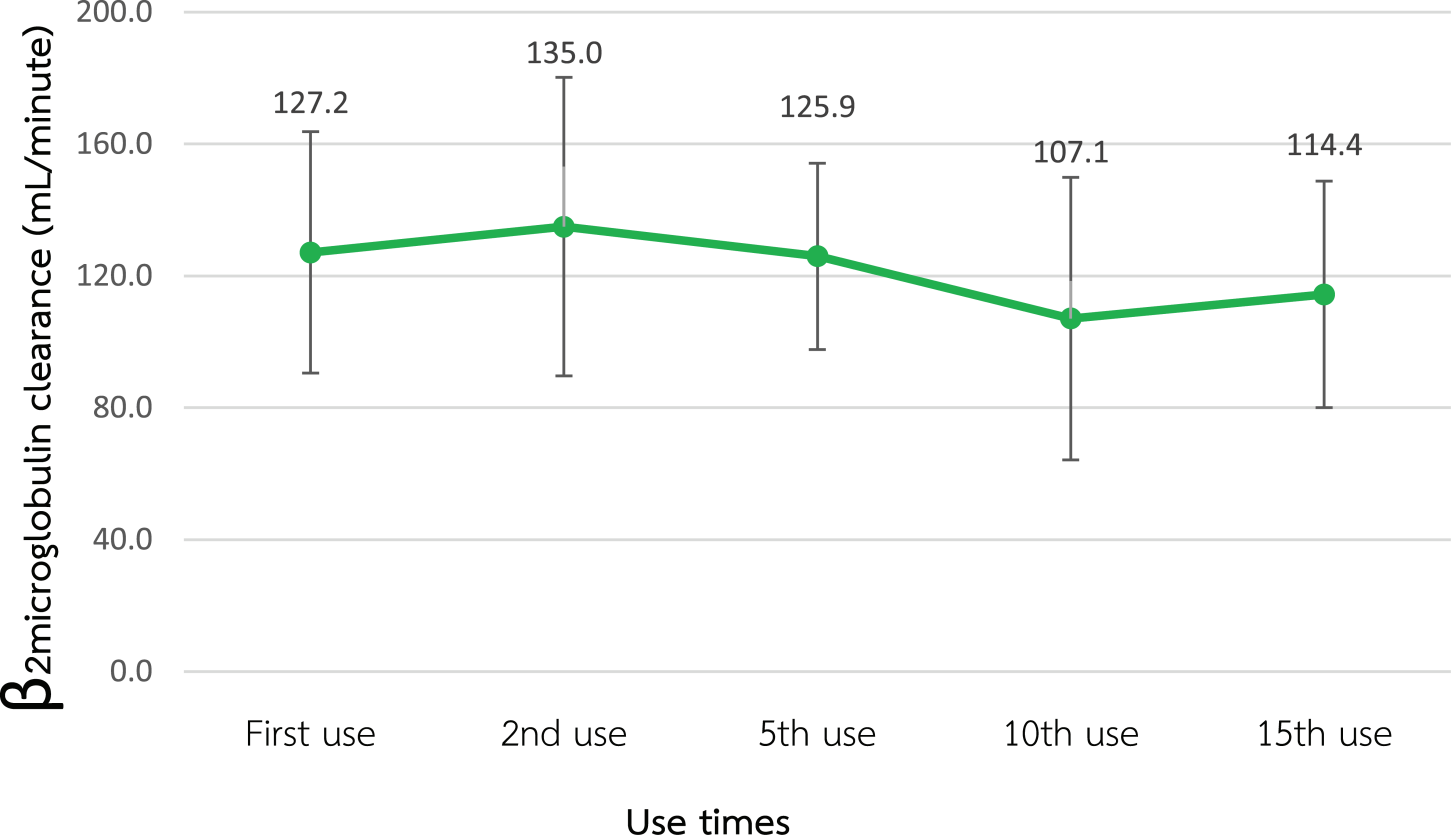
Change in β2-microglobulin clearance after the reuse of super high-flux dialyzers (*n* = 15). There was no statistically significant difference between the clearance of the first use and that of repeated uses.

**FIGURE 3:**
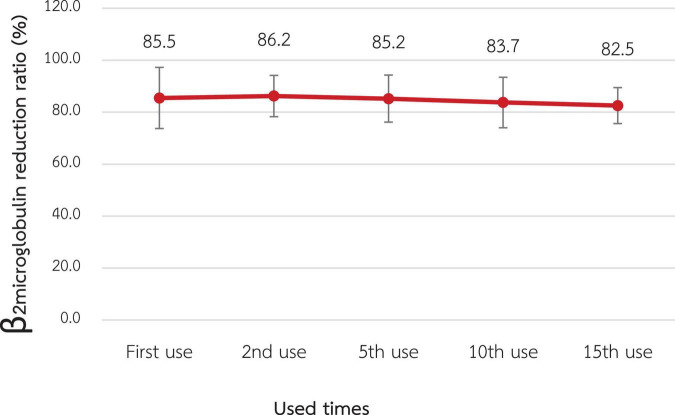
Change in the reduction ratio of β2-microglobulin after reuse of super high-flux dialyzers (*n* = 15). There was no statistically significant difference between the clearance of the first use and that of repeated uses.

According to Figures 2, 3, the mean β2M clearance of the 1st use was 127.2 ± 18.3 mL/min, which was not significantly different from the 2nd, 5th, 10th, and 15th use (135.0 ± 22.6, 126.0 ± 14.1, 107.1 ± 21.4, and 114.4 ± 17.2 mL/min, respectively). Additionally, the RR of β2M was comparable from the 1st use to the 2nd, 5th, 10th, and 15th use (85.5% ± 5.9%, 86.2% ± 4.0%, 85.2% ± 4.5%, 83.7% ± 4.9%, and 82.5% ± 3.5%, respectively).

### α1M removal

**FIGURE 4:**
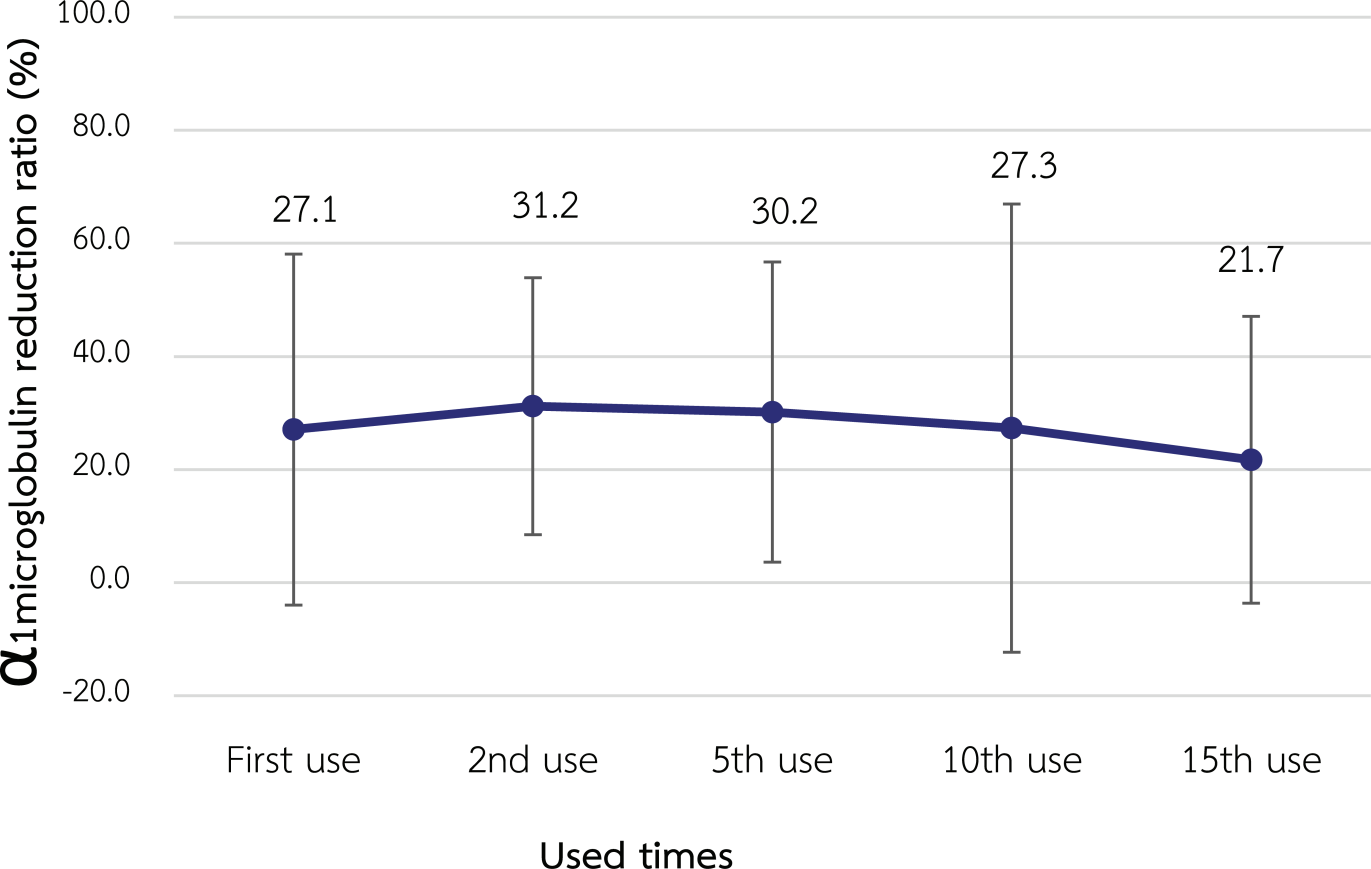
Change in the reduction ratio of α1-microglobulin after reuse of super high-flux dialyzers (*n* = 15). There was no statistically significant difference between the clearance of the first use and that of repeated uses.

The α1M RR for the 1st use was 27.1% ± 15.5% which was not statistically significantly different from the RR of the 2nd, 5th, 10th, and 15th use (31.3% ± 11.0%, 30.2% ± 13.3%, 27.3% ± 19.8% and 21.7% ± 12.7%, respectively) as shown in Figure 4.

### λ-FLC removal

**FIGURE 5:**
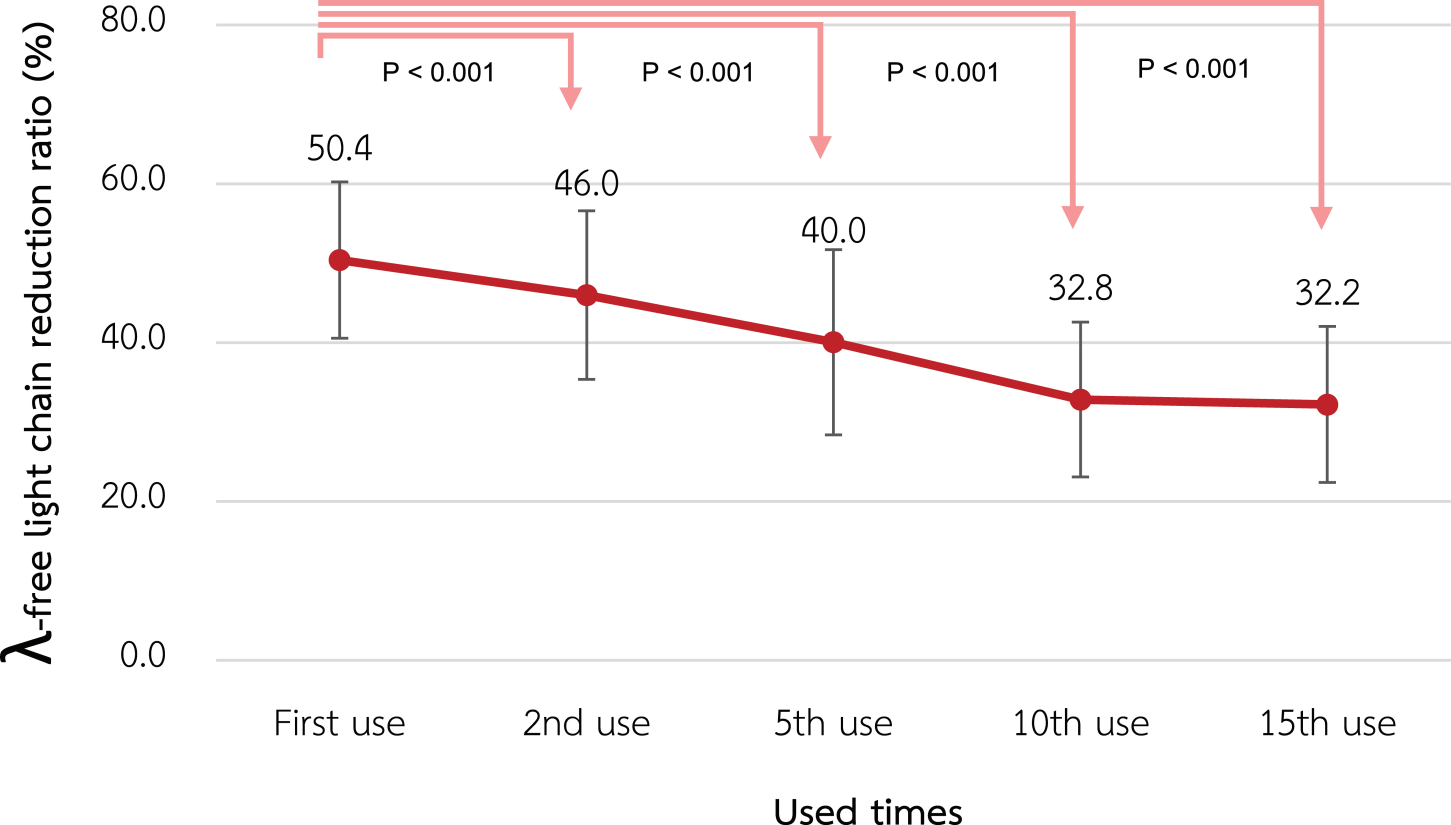
Change in the reduction ratio of the λ-free light chain after reuse of super high-flux dialyzers (*n* = 15). Compared to the baseline level, there were significant reductions in the λ-free light chain reduction ratio from the 2nd reuse to the 15th reuse.

The λ-FLC RR of the 1st use was 50.4% ± 4.9% and significantly decreased to 46.0% ± 5.3%, 40.0% ± 5.8%, 32.8% ± 4.9%, and 32.2% ± 4.9% at the 2nd, 5th, 10th, and 15th use, respectively (*p*-value < 0.001 for all pairwise comparisons) as shown in Figure 5.

### Indoxyl sulfate removal

**FIGURE 6:**
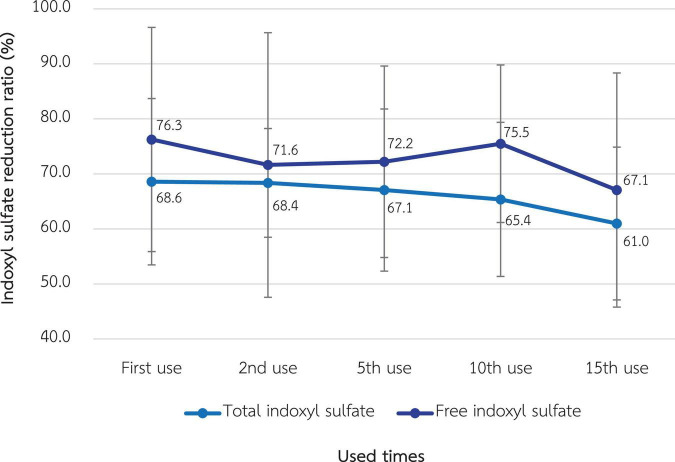
Change in the reduction ratio of total and free indoxyl sulfate after the reuse of super high-flux dialyzers (*n* = 15). There was no statistically significant difference between the clearance of the first use and that of repeated uses.

As shown in Figure 6, the RR of total and free indoxyl sulfate at the 1st use was 68.6% ± 7.6% and 76.3% ± 10.2%, respectively. There was no statistically significant difference in these RRs in the 15th use, which were 61.0% ± 6.9% and 67.1% ± 10.6% for total and free indoxyl sulfate, respectively.

### Albumin loss in dialysate and serum albumin

**FIGURE 7:**
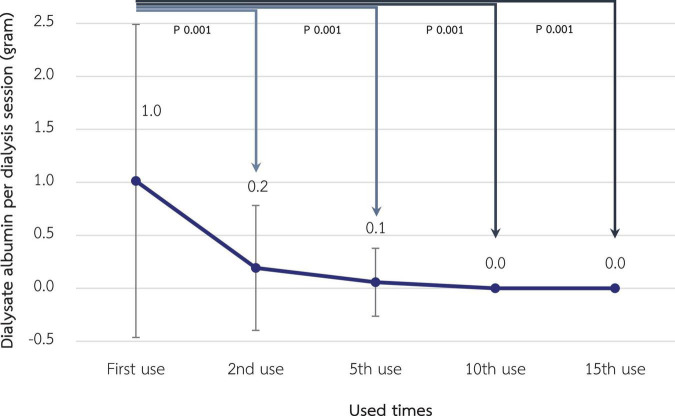
Change in dialysate albumin loss during hemodialysis session after reuse of super high-flux dialyzers (*n* = 15). Compared to the baseline level, there were significant reductions in dialysate albumin from the 2nd reuse, reaching the almost absence of albumin in the dialysate in the 10th and 15th reuse. This reflects the reduced albumin loss from expanded hemodialysis using reused super high-flux dialyzers.

According to Figure 7, albumin loss in dialysate decreased significantly from 1.01 grams at the 1st use to 0.19, 0.06, and <0.01 g at the 2nd, 5th, and 10th use, respectively (*p*-value < 0.001 for all pairwise comparisons). However, serum albumin of the included patients, which was 3.90 ± 0.22 g/L at baseline, was not significantly different in the following 3 months (3.95 ± 0.24, 3.92 ± 0.26, and 3.88 ± 0.22 g/L, respectively). Albumin and other small solutes, along with Kt/V, are shown in Table 3.

**TABLE 3:** Change in laboratory parameters of included patients (*n* = 5) before (baseline) and after receiving hemodialysis with reused super high-flux dialyzers ELISIO-21HX.

Parameter	Value in mean ± SD			
	Baseline	Month 1	Month 2	Month 3
Kt/V	2.8 ± 0.8	2.6 ± 0.8*	2.7 ± 0.7	2.9 ± 0.8
URR (%)	88.2 ± 4.7	86.0 ± 5.6*	87.4 ± 4.6	89.3 ± 4.7
nPCR (g/d)	1.2 ± 0.4	1.3 ± 0.2	1.4 ± 0.3*	1.4 ± 0.3*
Serum albumin (g/L)	3.9 ± 0.2	3.94 ± 0.2	3.92 ± 0.3	3.9 ± 0.2
Hemoglobin (g/dL)	11.7 ± 0.7	11.6 ± 0.5	11.4 ± 0.2	11.2 ± 1.4
Phosphate (mg/dL)	3.4 ± 1.2	3.6 ± 0.9	3.9 ± 0.9	3.3 ± 1.4
ESR (mm/h)	17.2 ± 13	19.6 ± 9.0	18.8 ± 1.2	15 ± 8.2

nPCR, normalized protein catabolic rate; ESR, erythrocyte sedimentation rate; URR, urea reduction ratio.

*P-value < 0.05 compared between the value of that time point and the baseline level.

### Relationship between total cell volume and dialyzer effectiveness

**FIGURE 8:**
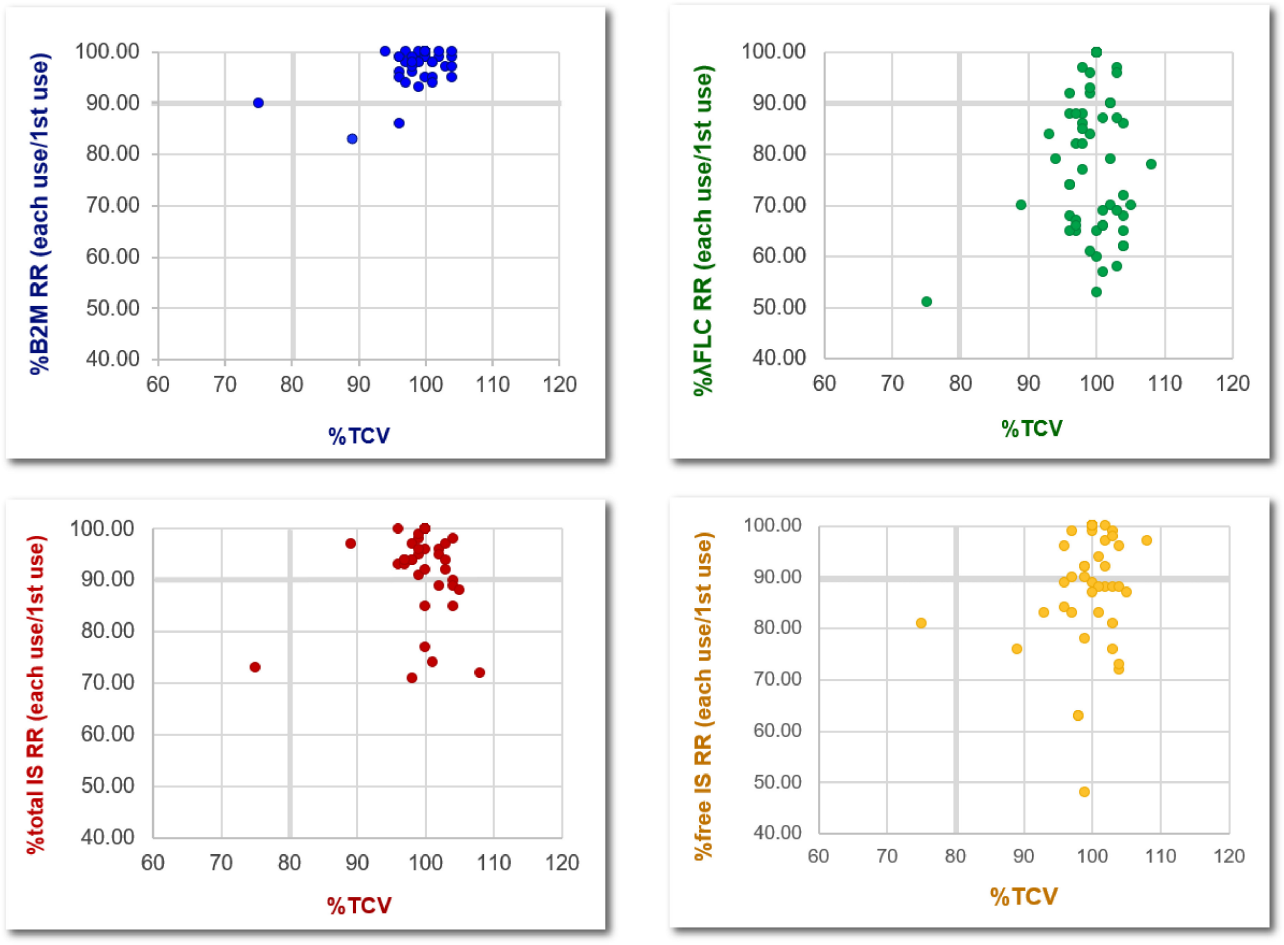
Relationship between the percentage changes, compared to the baseline level, of total cell volumes and the removal of various middle-molecule uremic toxins after the reuse of super high-flux dialyzers (15 dialyzers, with 4 repeated measurements in the 2nd, 5th, 10th, and 15th reuses). B2M, β2-microglobulin; λFLC, λ-free light chain; IS, indoxyl sulfate; RR, reduction ratio; TCV, total cell volume.

As shown in Figure 8, dialyzer TCV > 80% of initial values after reprocessing with peracetic acid was associated with β2M removal of >90% of the initial value. However, this TCV cut point did not correlate with the change in the removal of α1M, λ-FLC, and indoxyl sulfate.

### Adverse events

There were no dialysis or infectious complications occurring throughout the study period. Dialyzer reprocessing could be performed without technical problems.

## Discussion

Our study is the first to examine the effectiveness of middle-molecule uremic toxin removal by SHF after conventional reprocessing with peracetic acid (18). Although there was no impairment of the small solute clearance from SHF reuse as reflected by TCV higher than 80% until the last permitted reuse time, middle-molecule uremic toxins removal needs more attention for the high-flux dialyzer and SHF, since this is the focus of recent dialysis techniques.

An accumulation of β2M, a small middle-molecule uremic toxin, is associated with mortality and morbidity (19, 20). Indeed, augmented β2M clearance is an important characteristic of this novel dialyzers (21). HDx, an HD using SHF, has been shown to achieve a comparable β2M removal and a higher removal of larger uremic toxins, such as α1M and λ-FLC, compared to the high volume online HDF (8, 13). Although HDx is more feasible for most dialysis centers to apply compared to online HDF, the high cost of SHF could hamper widespread implementation. The plausibility of reusing SHF, which is originally designated as single use, without an unacceptable decrease in dialyzer performance could theoretically overcome this obstacle, since dialyzer reuse has been shown to reduce the cost of dialysis by 15%–30% and may pose an environmental benefit due to reducing medical waste from the dialyzer discard (22, 23). Unlike the reprocessing of high-flux dialyzer that has been shown to reduce β2M removal by up to 60% (24, 25), SHF reprocessing in our study does not significantly impair the RR of both β2M and α1M. Moreover, all mean β2M RR is still higher than 80% for all reuses, which is comparable to the RR achieved by high volume online HDF in previous studies that demonstrated the clinical benefit of such technique (7, 13). Therefore, we suggest this cut point of β2M RR as a checklist to discard SHF after reuse. Regarding α1M, another small middle-molecule uremic toxin, the optimal RR derived from a previous study of online HDF and HDx in our study is less rigid and is around 25%–30% (26).

Considering larger middle-molecule uremic toxins, the RR of λ-FLC significantly decreased since the second reuse and reached 40% since the 5th reuse. The assumption is that the size of λ-FLC is close to the pore size of the SHF. Therefore, after reprocessing the dialyzer with peracetic acid, the size of some pores decreases to be smaller than the size of λ-FLC due to the remaining protein on the dialyzer membrane. The RR of λ-FLC achieved by online HDF from previous studies is around 40% (5, 27, 28). This might limit the reuse time of SHF to not exceed 5 times in further practice, as suggested in Table 4.

**TABLE 4:** Suggested reuse protocol for super high-flux dialyzers based on the results of our study.

Protocol characteristics	Detail
Dialyzer model	ELISIO-21HX
Optimal reuse time	5 (not exceed 15)
Reprocessing method	Peracetic acid disinfection
**Criteria for discarding dialyzer**	
Total cell volume (TCV)	<80% of baseline level
β2-microglobulin reduction ratio	<80% (absolute number)
α1-microglobulin reduction ratio	<25%–30% (absolute number)
λ**-**free light chain reduction ratio	<40% (absolute number)
Free indoxyl sulfate reduction ratio	<56% (absolute number)
Dialysate albumin loss	>1–3 g per session
Observed clot in the dialyzer fiber	
Unexplained hypoalbuminemia	

Regarding protein-bound uremic toxins, such as indoxyl sulfate, there was a trend in decline of RR after reuse, although not statistically significant, in our study. This pattern was similar for both total and free indoxyl sulfate levels. Data from a previous study in online HDF suggested the optimal RR for free indoxyl sulfate of 56% (26). A major concern for HDx is that the loss of dialysate albumin is greater than that of the HDF, possibly resulting in transient hypoalbuminemia. According to previous research, the median loss of albumin per HDx session is approximately 3 g (29). In our study, albumin loss in the dialysate with SHF was 1.01 g initially, which was significantly reduced to almost none after reprocessing. This could be due to the smaller pore size of the membranes after exposure to peracetic acid. Ameliorating albumin loss via HDx might be the advantage of reusing SHF and correlated with the finding that serum albumin, along with other inflammatory and nutritional markers, in the patients who received HDx for 3 months was not significantly altered.

Reuse of dialyzers may be more cost-effective compared to a single-use strategy if the cost associated with dialyzer manufacturing is more expensive than the reprocessing or labor costs. In developing countries, evidence has shown that conventional dialyzer reuse can reduce the cost of hemodialysis by 30%–70% per session (23, 30). Therefore, dialyzer reuse may improve patient access to hemodialysis and increase the opportunity to use high-quality dialyzers, especially SHF, without compromising the economic and environmental burdens. According to our study, HDx with reused SHF is a plausible and safe novel technique. The limitations included the small sample size and the restriction to only short-term outcomes. The specific dialyzer type and reprocessing protocol used in our study, which were polyester sulfone and peracetic acid, respectively, require cautious generalizability to other reprocessing techniques or dialyzer membranes. Moreover, adverse events from the reuse process should be monitored upon long-term use in various centers. Further studies are required to explore the clinical benefits, environmental issues, economic burden, and cost-effectiveness of different dialysis techniques such as conventional HD, online HDF, HDx with single-use SHF, and HDx with reuse protocol.

## Conclusion

Reprocessing of SHF for reuse up to 15 times did not significantly reduce the RR of β2M and α1M. λ-FLC RR was significantly reduced after the 2nd reuse and fell below the optimal level reported by previous studies since the 5th reuse. Albumin loss during dialysis was significantly reduced after reuse, along with the trend in the reduction of RR of protein-bound indoxyl sulfate. Nevertheless, serum albumin from the included patients remained stable. HDx with reused SHF could be an alternative to online HDF and HDx with single-use SHF to lower the cost, reducing the medical waste associated with dialyzer discard, while maintaining the modern dialysis efficacy.

## Data Availability

The raw data supporting the conclusions of this article will be made available by the authors, without undue reservation.
