# Supplementary material for: Plausibility of super high-flux dialyzer reuse in maintenance hemodialysis
Source: Front Med (Lausanne). 2025 Oct 22;12:1655099. doi: 10.3389/fmed.2025.1655099 (PMC12586166; doi:10.3389/fmed.2025.1655099)
Supplement: Supplementary file 1 [file Data_Sheet_1.pdf]

Supplementary Material for "Plausibility of Super High-flux Dialyzer Reuse in Maintenance Hemodialysis"

Piyapun Prapunwatana[1,2], Thana Thongsricome[1,3], Asada Leelahavanichkul[4], Patcharin Injan[1], Pajaree Chariyavilaskul[5], Paweena Susantitaphong[1], Yingyos Avihingsanon[1], Somchai Eiam-Ong[1], Khajohn Tiranathanagul[1]

[1] Division of Nephrology, Department of Medicine, Faculty of Medicine, Chulalongkorn University, Bangkok, Thailand

[2] Department of Medicine, Chulabhorn Hospital, Chulabhorn Royal Academy, Bangkok, Thailand

[3] Department of Physiology, Faculty of Medicine, Chulalongkorn University, Bangkok, Thailand

[4] Center of Excellence in Translational Research in Inflammation and Immunology, Faculty of Medicine, Chulalongkorn University, Bangkok, Thailand

[5] Center of Excellence in Clinical Pharmacokinetics and Pharmacogenomics, Department of Pharmacology, Faculty of Medicine, Chulalongkorn University, Bangkok, Thailand

Study protocol for the reuse of super high-flux dialyzers ELISIO-21 HX

After completion of hemodialysis and disconnecting the dialyzer from the dialysis circuit, transfer the dialyzer to the reprocessing area and reprocess following these steps:

1. Manual rinsing of the dialyzer with reverse osmosis (RO), from a dialysis water loop, until no visible blood and clots remained. Specialized devices from the manufacturer were used to rinse the RO water in the blood inlet to clean the blood clot in the hollow fibers without dissociating the header from the dialyzer body.

1A 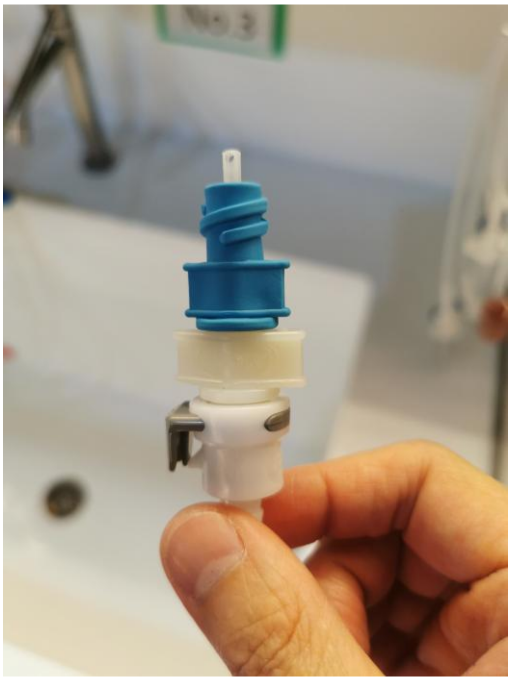

1B 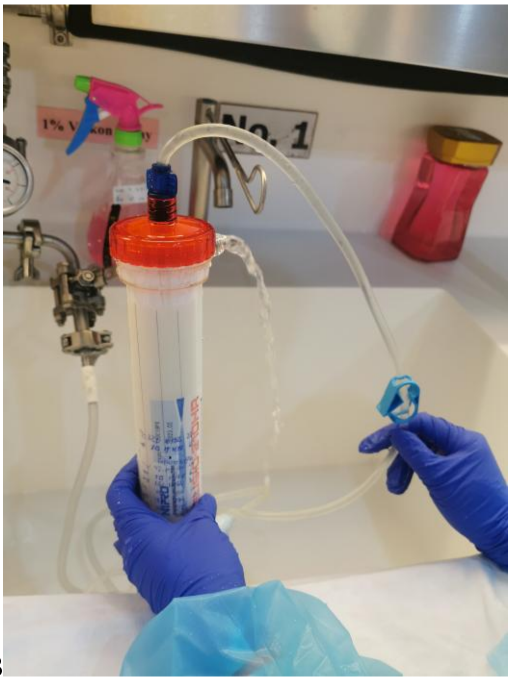

Figure S1A Specialized devices to connect the reverse osmosis water from the dialysis water loop to the blood inlet of the dialyzer

Figure S1B Rinsing process with reverse osmosis water entering the dialyzer through the blood inlet

2. Cleaning and sterilization of the dialyzer with the automated machine KIDNY-KLEEN™ Dialyzer Reprocessor, Model Compact II (Meditop Co., Ltd., Thailand) using 5% peracetic acid. Since our center routinely uses automated reprocessing machines, no manual cleaning-sterilizing protocol was developed. However, the manual process could theoretically be applied since this step is similar to that of the conventional dialyzer reprocessing.

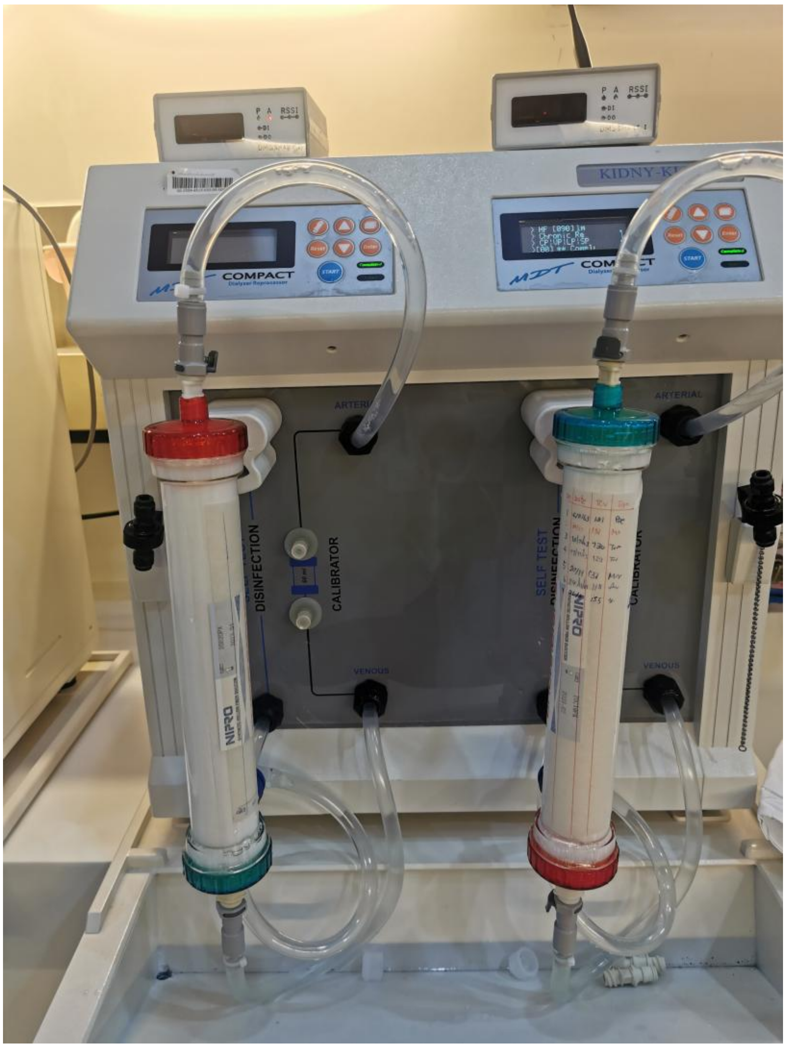

Figure S2 Automated dialyzer reprocessing machine. After connecting the blood inlet and the blood outlet to the machine and pressing the button, cleaning, measurement of total cell volume, and sterilizing with 5% peracetic acid will automatically be performed in about 10 minutes per dialyzer.

3. Testing of the total cell volume (TCV) of the dialyzer, which could be performed by the automated reprocessing machine immediately after the cleaning process. This should be done manually as the conventional dialyzer reuse in the case of manual reprocessing. Dialyzers were discarded if the TCV decreased to less than 80% of the baseline value, according to the recommendation by the Kidney Disease Outcomes Quality Initiative (KDOQI) guidelines 2006.

4. Storage of the sterilized dialyzer for at least 11 hours, to allow adequate time for sterilization, until the next use.
5. For subsequent use, adequate rinsing of the dialyzer with normal saline solution for at least 15 minutes was recommended to ensure that there was no residual peracetic acid remaining in the dialyzer. However, peracetic acid is easily dissociated and mainly does not enter the patient's circulation like other sterilizing agents.
